# A Small Motor Cortex Lesion Abolished Ocular Dominance Plasticity in the Adult Mouse Primary Visual Cortex and Impaired Experience-Dependent Visual Improvements

**DOI:** 10.1371/journal.pone.0137961

**Published:** 2015-09-14

**Authors:** Justyna Pielecka-Fortuna, Evgenia Kalogeraki, Franziska Greifzu, Siegrid Löwel

**Affiliations:** Department of Systems Neuroscience, Johann-Friedrich-Blumenbach-Institut für Zoologie und Anthropologie and Bernstein Fokus Neurotechnologie, Georg-August-Universität Göttingen, Göttingen, Germany; University of Montreal, CANADA

## Abstract

It was previously shown that a small lesion in the primary somatosensory cortex (S1) prevented both cortical plasticity and sensory learning in the adult mouse visual system: While 3-month-old control mice continued to show ocular dominance (OD) plasticity in their primary visual cortex (V1) after monocular deprivation (MD), age-matched mice with a small photothrombotically induced (PT) stroke lesion in S1, positioned at least 1 mm anterior to the anterior border of V1, no longer expressed OD-plasticity. In addition, in the S1-lesioned mice, neither the experience-dependent increase of the spatial frequency threshold (“visual acuity”) nor of the contrast threshold (“contrast sensitivity”) of the optomotor reflex through the open eye was present. To assess whether these plasticity impairments can also occur if a lesion is placed more distant from V1, we tested the effect of a PT-lesion in the secondary motor cortex (M2). We observed that mice with a small M2-lesion restricted to the superficial cortical layers no longer expressed an OD-shift towards the open eye after 7 days of MD in V1 of the lesioned hemisphere. Consistent with previous findings about the consequences of an S1-lesion, OD-plasticity in V1 of the nonlesioned hemisphere of the M2-lesioned mice was still present. In addition, the experience-dependent improvements of both visual acuity and contrast sensitivity of the open eye were severely reduced. In contrast, sham-lesioned mice displayed both an OD-shift and improvements of visual capabilities of their open eye. To summarize, our data indicate that even a very small lesion restricted to the superficial cortical layers and more than 3mm anterior to the anterior border of V1 compromised V1-plasticity and impaired learning-induced visual improvements in adult mice. Thus both plasticity phenomena cannot only depend on modality-specific and local nerve cell networks but are clearly influenced by long-range interactions even from distant brain regions.

## Introduction

Brain plasticity and learning are especially important after a brain lesion, such as a stroke, to enable rehabilitation. A stroke has not only a detrimental effect on the directly affected tissue, but it can also impact more distant regions and to some extent even the contralateral hemisphere, causing a transhemispheric diaschisis [1, 2]. Little is understood, however, about network and long-range influences on brain plasticity and learning-induced changes in sensory responses. A well-established model system to study experience-dependent neuronal plasticity is ocular dominance (OD) plasticity in the primary visual cortex (V1) [3–5]. In a previous study, we showed that a photothrombotic lesion in primary somatosensory cortex (S1) completely abolished OD-plasticity in the binocular zone of V1, and also abolished sensory learning in adult mice: neither the spatial frequency threshold (“visual acuity”) nor the contrast threshold (“contrast sensitivity”) of the open eye improved after MD and daily testing in the optomotor setup [6]. To investigate whether plasticity impairments from outside V1 are spatially restricted or can also be triggered from more distant brain regions, we induced a small lesion in the motor cortex region M2. We then analyzed the impact of this lesion on two different plasticity paradigms, induced by a 7 day MD of 3-month-old mice: (i) the learning-induced enhancement of the visual acuity and contrast sensitivity thresholds of the optomotor reflex of the open eye, measured by a behavioral optomotry task [7], and (ii) OD-plasticity in V1 visualized by *in vivo* optical imaging of intrinsic signals [8, 9]. In contrast to sham-treated control mice, mice with a distant, small stroke lesion in M2 did not show an OD-shift towards the open eye in V1 of the lesioned hemisphere. While OD-plasticity was abolished in the lesioned hemisphere it remained present in the nonlesioned hemisphere, alluding to a more specific disturbance and arguing against some “whole-brain” sickness preventing plasticity. Moreover, the learning-induced increases in both “visual acuity” and “contrast sensitivity” of the open eye were severely impaired in the lesioned animals. Thus, even a small, superficial and distant cortical lesion can affect plasticity in V1 of the same hemisphere and impair learning-induced visual improvements in adult mice.

## Material and Methods

All experimental procedures were performed according to the German Law on the Protection of Animals and permitted by the local government: Niedersächsisches Landesamt für Verbraucherschutz und Lebensmittelsicherheit (permission number 33.9-42502-04-10/0326).

### Animals

C57BL/6J mice (PD74-110 at the day of the optical imaging experiment) were obtained from the mouse colony of the central animal facility of the University Medical Center, Göttingen, Germany, and housed in an animal room with a 12-h light/dark cycle, with food and water available ad libitum.

### Induction of photothrombosis (PT)

A photothrombotic lesion was induced in the left motor cortex region M2, 3mm anterior to the anterior border of V1, by using the Rose Bengal technique introduced by Watson et al. [10]. As described previously [6], mice were initially anesthetized with 2% isoflurane, and anesthesia was maintained with 1% isoflurane. The animals were placed in a stereotaxic frame and body temperature was maintained at 37°C. The skin above the skull was incised and an optic fiber bundle (aperture: 1.0mm), mounted on a cold light source (Schott KL 1500), was positioned 2mm anterior to the bregma and 1mm lateral to the midline. Next, 100μl Rose Bengal (Aldrich; 10mg/ml in 0.9% NaCl) was injected intravenously. After 5min, the illumination period of 15min was started. The skin was sutured and the animals recovered in their cage. No behavioral effects were observed. All sham animals were treated identically but the light source was not switched on (control group).

### Monocular deprivation (MD)

The right eye was deprived for 7 days according to published protocols [9, 11, 12]. Animals were checked daily to make sure that the eyes remained closed.

### Visual acuity and contrast sensitivity

Both the spatial frequency threshold (“visual acuity”) and the contrast threshold (“contrast sensitivity”) of the optomotor reflex of all mice was measured daily using the optomotor system of Prusky et al. [7] as described previously [6, 13]. All animals were first tested in the optomotor setup during 7 days of MD (or no-MD) and then their visual cortical activity was visualized using intrinsic signal optical imaging.

### Optical imaging of intrinsic signals and visual stimuli

After the behavioral tests, mouse visual cortical responses were recorded and analyzed as described previously [6].

#### Surgery

Briefly, mice were box-anesthetized with 2% halothane in O_2_:N_2_O (1:1) and injected with atropine (0.3mg/mouse s.c.; Franz Köhler), dexamethasone (0.2mg/mouse s.c.; Ratiopharm), and chlorprothixene (0.2mg/mouse i.m.; Sigma). After placing animals in a stereotaxic frame, anesthesia was maintained with 0.8% halothane in a 1:1 mixture of O_2_:N_2_O.

#### Data acquisition and visual stimulation

Mouse V1-responses were recorded through the skull using the “Fourier”-imaging method of Kalatsky and Stryker [8] and optimized for the assessment of OD-plasticity [9]. V1-signals were visualized with a CCD-camera (Dalsa 1M30) using a 135x50mm tandem lens configuration (Nikon), with red illumination light (610±10nm). Active brain regions absorb more of the red light and appear darker in the images. Frames were acquired at a rate of 30Hz, temporally binned to 7.5Hz, and stored as 512x512 pixel images after spatial binning of the camera image.

Visual stimuli were presented on a high refresh rate monitor (Hitachi, ACCUVUE, HM-4921-D, 21”) positioned 25cm from the eyes. Stimuli consisted of drifting white horizontal bars (2° wide) restricted to the binocular field of the recorded hemisphere (-5° to +15° azimuth for the left visual cortex), as described previously [13]. The amplitude component of the optical signal represents the intensity of neuronal activation (expressed as fractional change in reflectance x10^-4^) and was used to calculate OD. At least 3 maps per hemisphere were averaged to compute the ODI as: (C−I)/(C+I), with C and I representing the response magnitudes of each pixel to visual stimulation of the contralateral and ipsilateral eye.

### Perfusion and tissue processing

After optical imaging, all mice were perfused transcardially with 1% heparin in 0.9% NaCl for 2min followed by 4% paraformaldehyde (PFA, pH 7.4) for 3min. The brain was removed and postfixed in 4% PFA (pH 7.4) for one day and then transferred to cryoprotectant solution (10% sucrose, 20% glycerol). The brains were frozen in methylbutane and stored at -80°C. Coronal brain sections were cut on a sledge microtome at 40μm.

### Lesion analysis

To determine the size and location of the cortical PT-lesions, coronal brain sections were Nissl-stained and every third section was analyzed under a microscope with 10x objective (Axioskop, Carl Zeiss). Quantitative parameters were measured on digitized sections using AxioVision (40 4.8.2.0.).

### Statistical analyses

All intra- and intergroup comparisons were analyzed by a two-tailed Student's t-test. The intergroup comparison of the enhancement of visual acuity was analyzed by ANOVA with repeated measurements and Bonferroni correction. Pearson correlation was used to measure correlations of lesion parameters. The levels of significance were set as *P<0.05; **P<0.01; ***P<0.001. Data are represented as means±SEM.

## Results

### Location and size of the cortical lesion in motor cortex

**Fig 1:**
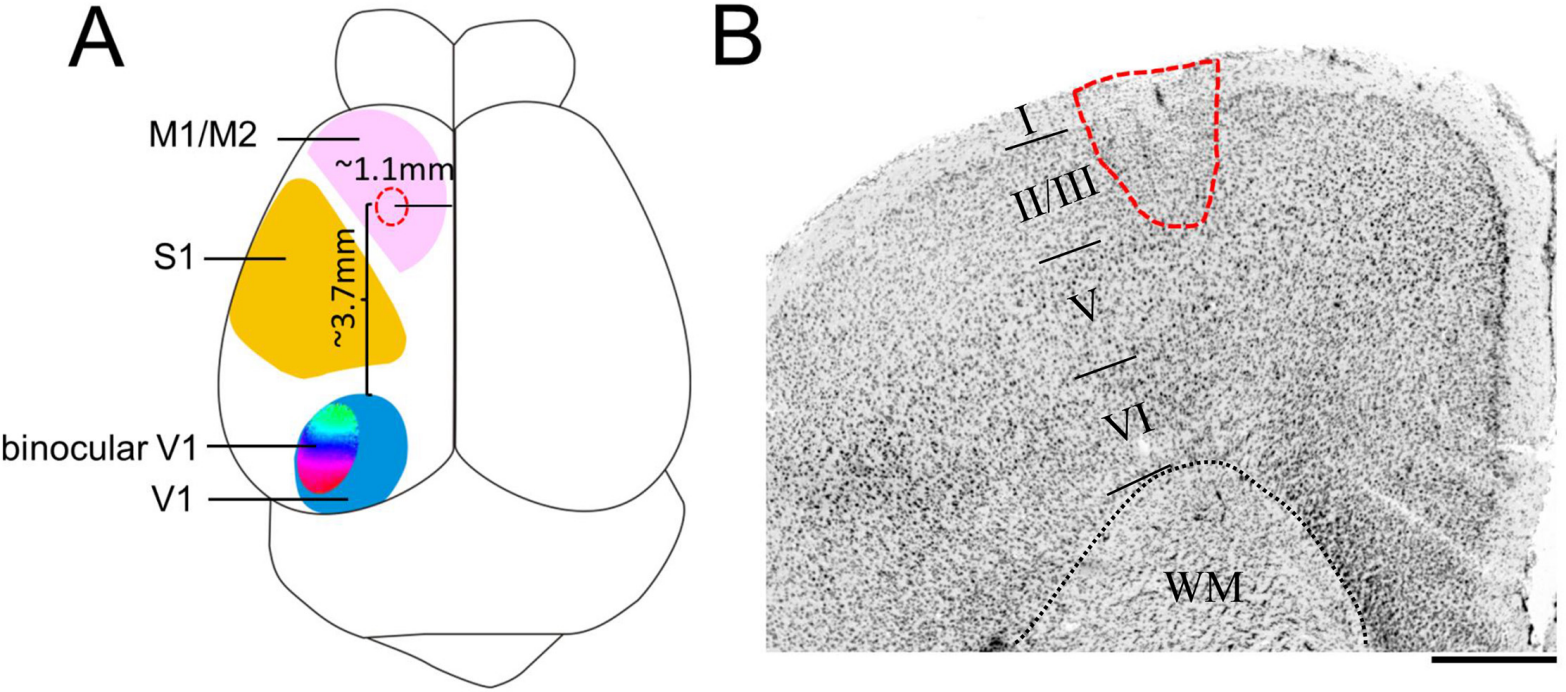
Localization and size of the cortical stroke. (A) Scheme of the average lesion location (red dotted circle/line) and size in the left motor cortex (M2) in respect to the primary visual cortex (V1) of a mouse brain. (B) Nissl-stained frontal section through a representative lesion, WM-white matter. Scale bar: 500μm.

PT-lesions were located in the left M2. Their average size was 0.6±0.01mm in medio-lateral and 0.7±0.01mm in anterior-posterior direction; the lesion center was located 3.7±0.1mm anterior to the anterior border of V1, 1.1±0.1mm lateral to the midline, 1.5±0.1mm anterior to the bregma and 0.3±0.04mm in depth, reaching into layers 2/3 (Fig 1). The average lesion volume was 0.12±0.03mm^3^. Notably, lesion size (volume) did not correlate with the enhancement of visual acuity (r = -0.544, P = 0.130) or the ODI (r = -0.356, P = 0.387) after MD. Likewise, there was also no correlation with the lesion location (distance of lesion end to V1) and the enhancement of visual acuity (r = -0.137, P = 0.725) or the ODI (r = -0.500, P = 0.207) after MD. These M2-lesions were on average smaller in diameter, depth (into the cortex) and volume, and further away from V1 compared to our previous PT-lesions in the primary somatosensory cortex (P<0.001 for all, t-test) [6].

### Cortical lesion in M2 impaired OD-plasticity in the lesioned hemisphere

**Fig 2:**
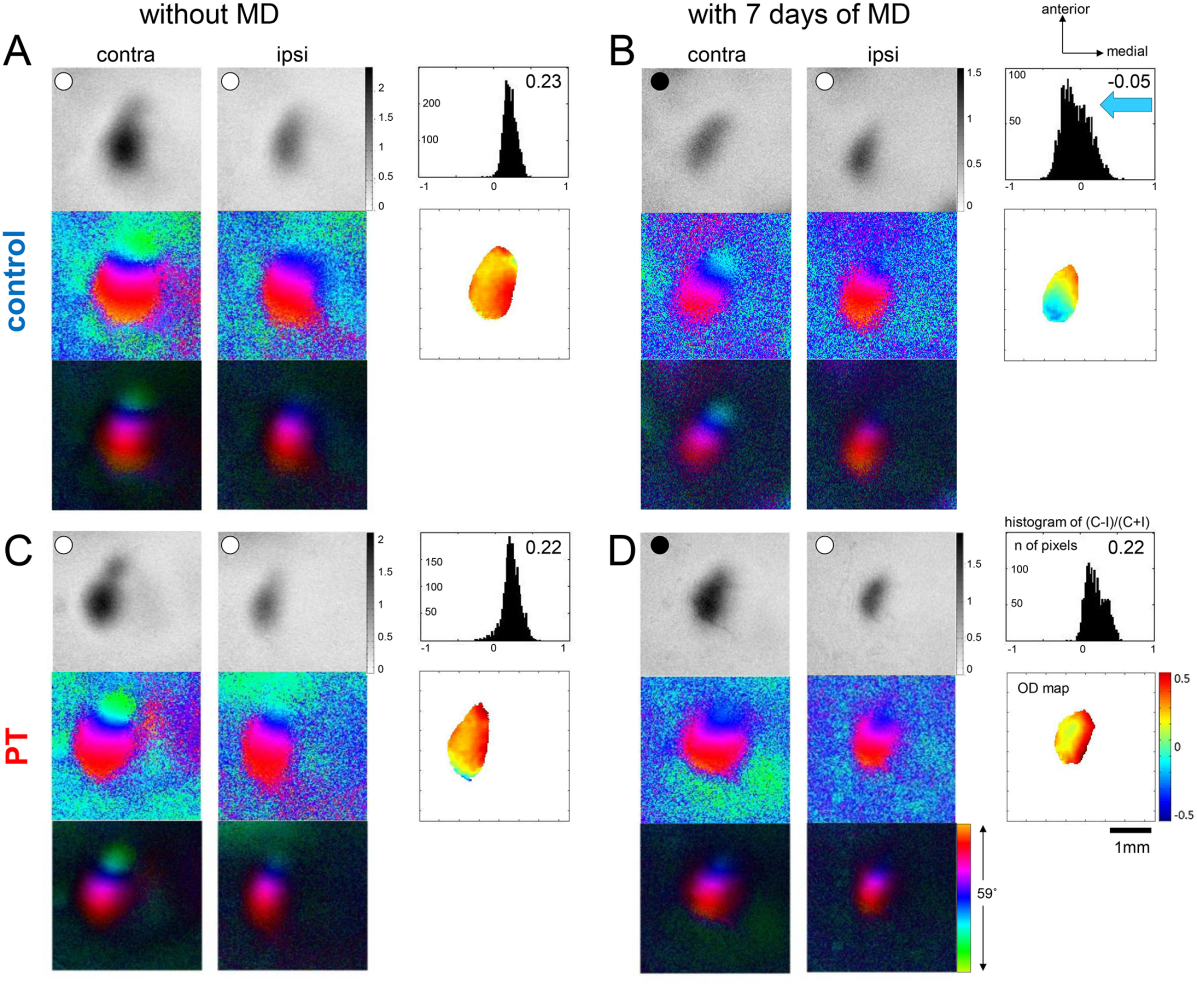
A cortical lesion in M2 impairs OD-plasticity in the visual cortex of the lesioned hemisphere. Optically recorded activity maps of the contralateral (contra) and ipsilateral (ipsi) eye in the binocular region of mouse primary visual cortex (V1) in sham-treated control mice (A, B) and in mice with a PT-lesion in M2 of the recorded hemisphere (C, D). Activity maps without MD are illustrated in the left column (A, C), maps after MD in the right column (B, D). Gray-scale coded response-magnitude maps and their quantification (top), and color-coded phase and polar maps of retinotopy (middle, bottom) are illustrated. The magnitude of the optical responses is illustrated as fractional change in reflection x10^-4^. For each experiment, the histogram of OD-scores (upper right), the OD-index (ODI), and the corresponding 2D OD-maps (ODI values are color-coded: red represents positive, blue negative values) are included. Note that without MD, activity patches evoked by stimulation of the contralateral eye were consistently darker than those after stimulation of the ipsilateral eye, 2D OD-maps display warm colors, and the average ODI is positive, indicating contralateral dominance (A, C). In contrast, MD for 7 days in control- (B) but *not* PT-animals (D) induced an OD-shift toward the open (ipsilateral) eye: the activity map of the ipsilateral eye was even darker than after contralateral (deprived) eye stimulation (black circle indicates MD eye), the histogram of OD-scores shifted to the left (blue arrow in B), the ODI was negative, and colder colors prevailed in the OD-map. In contrast, in PT-animals with MD (D), activity maps of the deprived (contra) eye remained darker than those of the open eye, the histogram of OD-scores and the average ODI were essentially unchanged and warm colors still prevailed in the OD-map.

**Fig 3:**
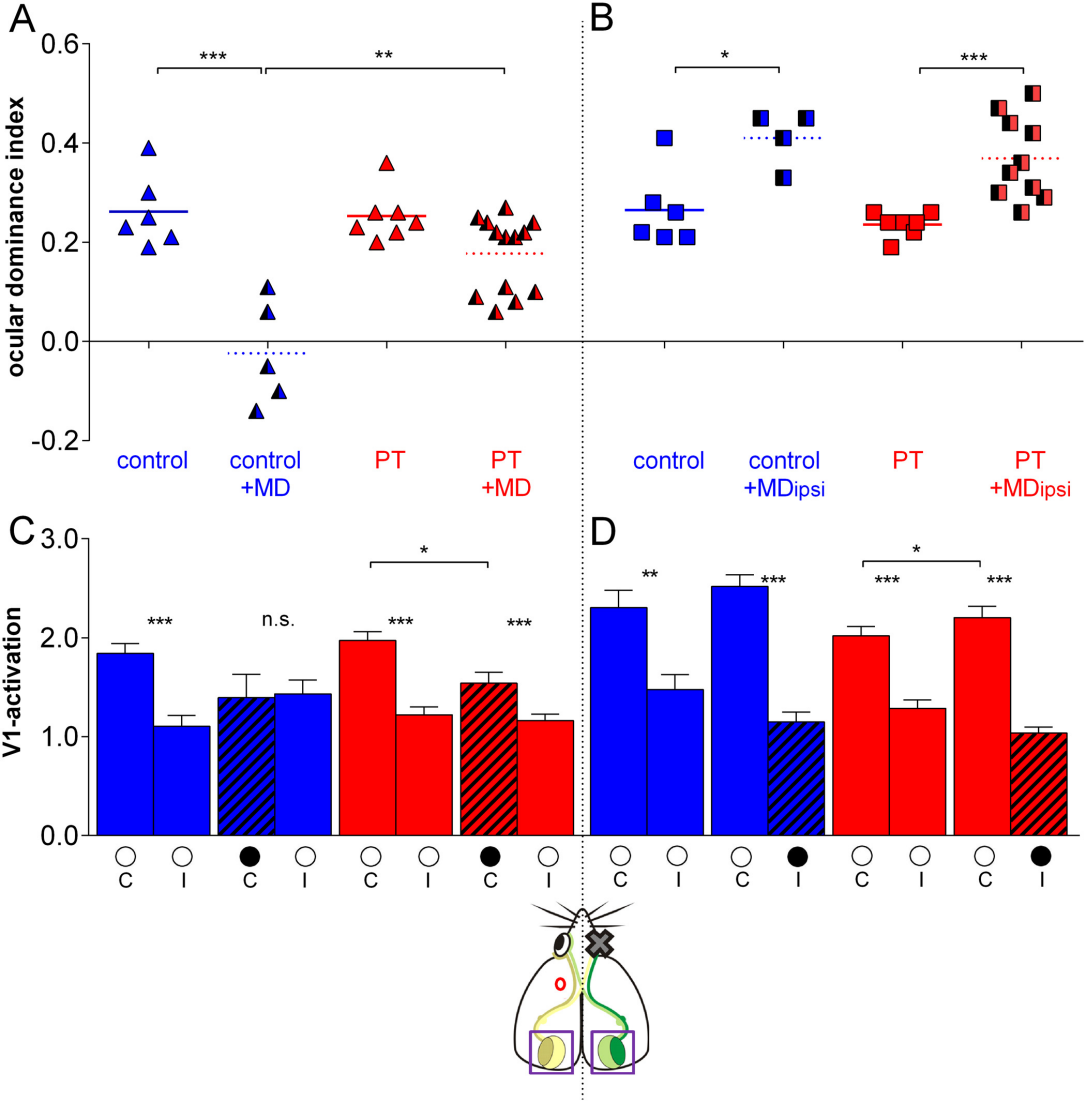
Quantification of the OD-indices and V1-activation. (A, B) Optically imaged OD-indices in sham-treated (control, blue) animals and after PT in M2 (red) without and with 7 days of MD of the right eye. Symbols represent ODI-values of individuals, means are marked by horizontal lines. (C, D) V1-activation elicited by stimulation of the contralateral (C) or ipsilateral (I) eye in control animals and after MD (black circle indicates MD eye). (A) In control mice, MD induced an OD-shift towards the open eye. In contrast, PT-mice did not show a significant OD-shift in the lesioned, left hemisphere. (B) In contrast, in the nonlesioned, right hemisphere, MD induced an OD-shift in PT-mice, as in control animals. (C, D) V1-activation after stimulation of the contra- and ipsilateral eye before and after MD in both control and PT-lesioned mice.

Visual cortical activity and plasticity was visualized using intrinsic signal optical imaging [8, 9]. In both control (sham-lesioned) and PT-lesioned mice without monocular deprivation (MD) visual stimulation of the contralateral eye induced stronger V1-activation than ipsilateral eye stimulation. Since more active brain regions absorb more of the red light with which the cortex was illuminated they appear darker in the activation maps (Fig 2A and 2C). To quantify the relative strength of V1-activation after visual stimulation of the left and right eye, we computed an OD-index (ODI), and this index is color-coded in the 2-dimensional OD-maps. In V1 of non-deprived mice, OD-maps of both groups displayed warm colors, indicating contralateral dominance, and the ODI-histogram was centered to the right of zero (Fig 2A and 2C). Seven days of MD in sham-treated mice induced an OD-shift towards the open eye, OD-maps were dominated by colder colors (Fig 2B) and the ODI decreased from 0.26±0.03 (n = 6) without MD to -0.02±0.05 (n = 5) after MD (P = 0.0005, t-test; Fig 3A). In contrast, in PT-lesioned animals, MD failed to induce an OD-shift because activity patches induced by visual stimulation of the deprived eye remained darker than those of the open eye: binocular visual cortex remained dominated by input from the contralateral eye, irrespective of whether this input was deprived or not (Fig 2C and 2D). The ODI of the PT-mice after MD was 0.18±0.02 (n = 13), and therefore *not* different from PT-mice without MD (0.25±0.02, n = 7, P = 0.067, t-test), but different from control animals after MD (P = 0.008, t-test; Fig 3A). Thus, even a small and distant cortical lesion abolished OD-plasticity in the lesioned hemisphere.

To test the inter-hemispheric influence of the PT-lesion, we also analyzed OD-plasticity in the nonlesioned hemisphere of the same animals. It was previously shown that MD of the ipsilateral (weaker eye) can result in an OD-shift in favor of the open, contralateral eye [14, 15]. In fact, MD of the ipsilateral eye induced such an increase in ODI in both the nonlesioned hemisphere of PT-mice, and in the control mice. In PT-mice, the ODI increased from 0.24±0.01 (n = 7) to 0.37±0.02 with MD (n = 10; P = 0.001, t-test; Fig 3B); in the sham-treated control mice, the ODI increased from 0.27±0.03 (n = 6) to 0.41±0.02 after MD (n = 4; P = 0.012, t-test; Fig 3B). The ODIs of control and PT-mice after MD were not different (P = 0.388, t-test). Thus, OD-plasticity was clearly present in the nonlesioned hemisphere of the PT-mice, indicating that the reduction of OD-plasticity in the lesioned hemisphere must be a rather hemisphere specific disturbance which does not impair the plasticity mechanisms in the entire brain.

We also compared the strength of V1-activation induced by visual stimulation of the contra- and ipsilateral eye in both hemispheres (Fig 3C and 3D). Confirming previous measurements in nondeprived mice [14, 15], V1-activation was significantly higher after visual stimulation of the contralateral (C) compared to the ipsilateral (I) eye in the left hemisphere of control animals (C/I: 1.8±0.1/1.1±0.1; P = 0.0001, t-test, n = 6) and in the lesioned hemisphere of PT-mice (C/I: 2.0±0.1/1.2±0.1, n = 7; P = 0.0001, t-test), indicating that the PT-lesion did not interfere with sensory driven V1-activation. In control mice, after MD of the contralateral eye, V1-activation via the two eyes was no longer different (deprived/open: 1.4±0.2/1.4±0.1; P = 0.859, t-test, n = 5; Fig 3C). In the lesioned hemisphere of PT-mice, V1-activation after deprived-eye stimulation remained higher than after open-eye stimulation (deprived/open: 1.5±0.1/1.2±0.1, n = 13; P = 0.0001, t-test; Fig 3C). Thus, while there was a small reduction of deprived eye responses in the lesioned hemisphere of PT-mice (P = 0.017), the contralateral (deprived) eye still dominated V1, as in nondeprived mice.

In the nonlesioned (right) hemisphere of PT-mice, OD-shifts remained present and were mediated by decreases in closed eye responses in V1 (P = 0.024; Fig 3D): V1-activation induced by stimulating the contralateral (open) eye remained stronger, irrespective of whether the animal had an MD of the ipsilateral eye or not (control: C/I, 2.3±0.2/1.5±0.1, n = 6; control+MD: 2.5±0.1/1.2±0.1, n = 4; PT: 2.0±0.1/1.3±0.1, n = 7; PT+MD: 2.2±0.1/1.0±0.1, n = 10; P = 0.002 in control animals, P = 0.0001 in all other groups; Fig 3D).

### A cortical lesion in M2 impaired enhancement of vision after MD

**Fig 4:**
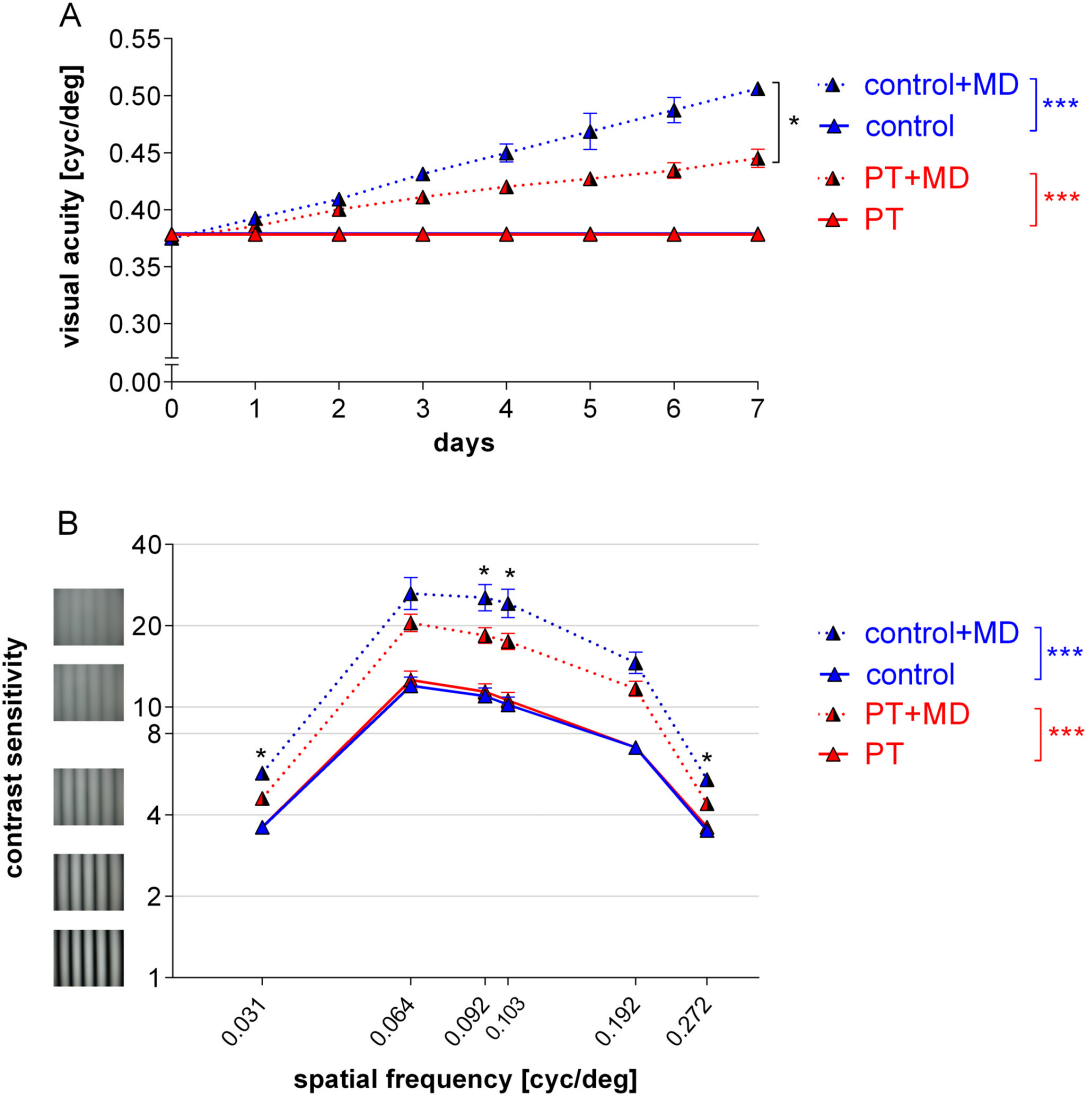
The M2-lesion severely impaired improvements of visual capabilities of the open eye after MD. Spatial frequency selectivity of the optokinetic response of the open eye in cycles per degree (cyc/deg) plotted against days (A) and contrast sensitivity at 6 different spatial frequencies (B) in control and PT-mice without and with MD (+MD). In both, control and PT-mice, values of the nondeprived (open) eye increased compared to mice without MD. However, the increase in PT-mice was only about 50% of the improvement of control mice.

We used the virtual‐reality optomotor setup [7] to determine visual acuity and contrast sensitivity thresholds of the optomotor reflex of mice of all experimental groups before and after MD (Fig 4A). In control animals, baseline visual acuity was 0.38±0.003 cyc/deg on day 0 and remained stable for 7 days without MD (P = 0.363, t-test, n = 6). After MD and daily testing, visual acuity of the open eye increased on average by 33±1% to 0.50±0.01 cyc/deg on day 7 (P = 0.00001, t-test, n = 5). Visual acuity development over the 7 days was different from control animals without MD (P = 0.0001, F_1,9_ = 86.72, ANOVA). While mice with a PT-lesion also showed an enhancement of visual acuity values of the open eye after MD, the average increase was only 19±2% (from 0.38±0.002 cyc/deg on day 0 to 0.45±0.008 cyc/deg on day 7; P = 0.000002, t-test, n = 13). Although this increase was different from PT-mice without MD (day 0: 0.38±0.002, day 7: 0.38±0.002, n = 8; P = 0.0001, F_1,19_ = 31.61, ANOVA), it was about 50% smaller than in control mice with MD (P = 0.015, F_1,16_ = 7.36, ANOVA).

We also measured the lowest contrast gratings at six different spatial frequencies that elicited an optomotor reflex in both control and PT-lesioned mice before and after MD (Fig 3B). Without MD, contrast sensitivities of control and PT-animals remained stable over time (control/PT: P>0.05, t-test, n = 6/8). After MD in control animals, contrast sensitivity (at 0.064 cyc/deg) of the open eye was enhanced by 62±8% (P = 0.0098, t test): it increased from 10±0.5 (corresponding to 10% contrast) to 26±4 (= 4% contrast; n = 5). In contrast, in PT-animals, MD enhanced contrast sensitivity of the open eye by only 29±4%: values increased from 12±0.7 (= 8% contrast) on day 0 to 21±2 (= 5% contrast) on day 7 (n = 13; P = 0.0003, t-test). Compared to control mice, the contrast sensitivity of the PT-mice after MD was lower at 4 spatial frequencies (0.031, 0.092, 0.192, 0.272cyc/deg; P<0.05, t-test). Likewise, the increase in contrast sensitivity after MD was reduced in PT- compared to control mice (P = 0.016 for 0.064cyc/deg, ANOVA).

Thus, the small PT-lesion in M2 measurably decreased the experience-induced improvements in both the spatial frequency and contrast thresholds of the optomotor reflex of the open eye after MD.

## Discussion

The aim of the present study was to examine the effect a small cortical lesion in the motor cortex area M2 on experience-induced plasticity in the primary visual cortex (V1). We observed that 3-month-old mice with a small lesion in M2 had impaired visual cortex plasticity and reduced sensory learning. In the lesioned mice, seven days of MD failed to induce the typical OD-shift observed in V1 of nonlesionend animals. In addition, lesioned mice showed an impaired improvement of the optomotor reflex of the open eye (“visual acuity” and “contrast sensitivity”). Besides the lack of experience-induced plasticity in the lesioned hemisphere, OD-shifts were still present in the nonlesioned hemisphere and similar in magnitude to the sham-lesioned control mice.

While lost experience-dependent plasticity in either V1 or S1 after a small cortical stroke lesion in the neighboring S1 or V1, respectively, has been previously observed [6, 16], it is possible that these inter-areal influences are restricted to immediately adjacent cortical regions. To test the range of these non-local influences on cortical plasticity, we induced the lesion in the anterior M2-region, at least 3mm anterior to the anterior border of V1, and thus more than 3 times further away than in the previous study [6]. Interestingly, the on average much smaller and more distant M2-lesions still impaired both OD-plasticity in V1 of the lesioned hemisphere and experience-induced visual improvements of the open eye. As previously observed after S1-lesions [6], OD-plasticity in the nonlesioned hemisphere was still present. The preserved OD-plasticity in the nonlesioned hemisphere clearly indicates a rather specific disturbance in the lesioned hemisphere and argues against some “whole-brain” sickness preventing plasticity in the entire brain. On the other hand, previous studies identified large-scale functional alterations in cortical activity of the nonlesioned contralateral hemisphere [17–20]. In addition, a recent *in vitro* study showed that electrophysiologically measured short-term plasticity after a lesion in V1 was abolished in V1 of both hemispheres, with even stronger effects in the contralateral hemisphere [20]. The differing findings can be most likely explained by homotopic versus heterotopic (our study) measuring sites with respect to the lesion location, different plasticity paradigm, lesion sizes as well as experimental conditions (*in vitro* vs. *in vivo*). Surprisingly, the small M2-lesion also impaired the experience-induced visual improvements after MD; however, they were not completely abolished as after the S1-lesions. Together these observations underline that not only local networks, but also long-range interactions might influence neuronal plasticity in a cortical region.

What long-range interactions might be responsible for the impaired visual plasticity? Recently, a functional coupling between M2 and V1 was observed [21] and optogenetic stimulation of M2 had a robust effect on V1-activation [22, 23]. Furthermore, anatomical tracing experiments revealed direct connections between V1 and auditory and somatosensory areas, as well as with motor and association cortices [24]. Although, the small M2-lesions did not measurably reduce basic V1-activation in our PT-lesioned mice, they prevented OD-plasticity, suggesting that stroke-induced disturbances in M2 have a modulatory influence on V1-plasticity. Thus, the direct connections between V1 and other sensory and motor cortices in rodents [24–27] suggest a complex and modulatory processing of multimodal information between different cortical regions.

Stroke leads to an overall imbalance of excitation and inhibition in the affected neuronal network [28], which—if untreated—can lead to negative consequences such as impaired plasticity and absent functional recovery. One of the major consequences of the ischemic damage leading to this imbalance is high level of the neurotransmitter glutamate, leading to excitotoxicity and ultimately neuronal cell death [29]. On the other hand, increased tonic inhibition in the peri-infarct zone of motor cortex (200μm lateral) was observed and reducing this excessive inhibition promoted functional recovery after stroke [30]. In contrast, a lesion induced in V1 led to reduced basal GABAergic transmission measured further away from the lesion border (~1mm lateral) and the same effect was observed in the nonlesioned hemisphere [20] suggesting that stroke-induced disturbances may be region as well as distance specific. Altogether these studies suggest that unphysiological changes in glutamatergic and GABAergic transmission after stroke can lead to negative consequences and thus may interfere with plasticity. There is some evidence suggesting that the excitatory/inhibitory balance may also be disturbed in V1 after a distant stroke. Several interventions showed that decreased cortical inhibition is a permissive factor for preserved OD-plasticity in V1 after an S1-lesion. Specifically, raising mice in an enriched environment not only preserved a low GABAergic inhibition and juvenile OD-plasticity into adulthood, but also protected from stroke induced impairments in V1 after an S1-lesion [13]. Furthermore, short-term dark exposure lowered cortical inhibition in rodents [31] and adult dark exposed mice continued to display OD-plasticity in V1 after an S1-lesion [32], as do young mice that have a low inhibitory tone in V1 [13]. Altogether, these studies implicate that a lower inhibitory tone protects V1 from OD-plasticity impairments after a stroke in S1. Whether also changes in excitatory circuitry may be beneficial for preserving V1-plasticity after a stroke in S1, and if similar mechanisms are true for the M2 lesions needs further investigation.

In addition to lost OD-plasticity after the M2-lesions we also observed a reduction in sensory learning. Compared to our previous PT-lesions in S1, the present M2-lesions were three times farther away and on average much smaller in diameter, depth and volume and extended only into the superficial cortical layers. Notably, compared to the S1-lesions, these smaller and more distant M2-lesions caused slightly smaller effects on the experience-induced improvements of both the visual acuity and contrast sensitivity thresholds of the optomotor reflex of the open eye. One possible interpretation is that the smaller and more distant lesions have caused a smaller inflammatory response. Inflammation is one of the major consequences of a stroke [33]. In the ischemic cortex, levels of pro-inflammatory mediators, including cytokines and adhesion molecules, increase about 1h after stroke, and return to basal values after 5 days [34, 35]. Thus, a milder inflammation could explain the smaller effect of our distant lesion. As we have shown previously, inflammation seems to be the major cause of the reduction in the experience-induced visual improvements after the small PT-lesions, because both anti-inflammatory drugs such as ibuprofen and a delay of 2 weeks between lesion-induction and the behavioural measurement rescued the visual improvements [6]. This interpretation was supported by our recent observation that mice raised in an enriched environment were less susceptible to stroke-induced impairments of visual plasticity [13]. In fact, EE housing reduced the increased inflammation level after a stroke [36]. Compared to standard-cage raised mice with a PT-lesion, which did not display any enhancement of vision after MD, enriched mice with a PT-lesion in S1 had a significant gain on baseline (acuity) through the open eye which was—however– 50% lower than in nonlesioned mice [13]. Thus raising mice in an enriched environment partially rescued this experience-induced “sensory learning”. Interestingly, about the same result was obtained here after the anterior PT-lesions: the visual improvements were about 50% of those of the nonlesioned sham-treated mice. It is therefore likely that the reduced sensory learning after MD in our PT-mice was also due to such a nonlocal inflammatory process.

The different effects of the PT-lesion on OD-plasticity vs. sensory learning (abolished vs. reduced) emphasizes that distinct neuronal subsystems underlie these two forms of visual plasticity as suggested before [6]. The enhancement of visual acuity and contrast sensitivity thresholds of the optomotor reflex of the open eye after MD is restricted to the monocular visual field, despite the dependence of the plasticity on binocular interactions [37]. In contrast, OD-shifts occur in the binocular region of V1 [9, 12, 38]. Moreover, OD-plasticity beyond the critical period mainly takes place in superficial cortical layers [39–41], while the enhancement of the optokinetic response involves the cortical control of the accessory optic system triggering the reflex, presumably from deep-layer efferents [37].

Our present finding that even a very small cortical lesion far away from V1 can impair V1-plasticity has potential medical relevance. In respect to that, post-mortem studies of patients diagnosed with dementia have identified cortical microinfarcts [42], which are thought to contribute to cognitive impairments and dementia [43]. In addition, the network consequences of stroke are increasingly appreciated: focal or widespread loss of blood flow caused an acute disruption of an individual’s connectome [44]. To summarize, visual plasticity phenomena cannot only depend on modality-specific and local nerve cell networks but are clearly influenced by long-ranging interactions even from distant brain regions. Nonlocal influences can thus strongly modulate the local networks’ function and plasticity.
